# Quantity Discrimination in Trained Lizards (*Podarcis sicula*)

**DOI:** 10.3389/fpsyg.2018.00274

**Published:** 2018-03-07

**Authors:** Maria Elena Miletto Petrazzini, Cristiano Bertolucci, Augusto Foà

**Affiliations:** ^1^Department of General Psychology, University of Padova, Padova, Italy; ^2^Department of Life Sciences and Biotechnology, University of Ferrara, Ferrara, Italy

**Keywords:** reptile cognition, lizard, *Podarcis sicula*, quantity discrimination, training procedure

## Abstract

Quantitative abilities have been reported in many animal species. Two main methods have been extensively used: spontaneous choice tests and training procedures. A recent study showed that ruin lizards are capable of spontaneously discriminating between the surface area of two food items of different size, but failed when food was presented in sets of discrete items differing in number. In the present study, we used a training procedure to further investigate quantitative abilities in ruin lizards. Subjects were presented with two sets of yellow disks differing either in number (Experiment 1) or in area (Experiment 2) and were trained on different discriminations of increasing difficulty (1 vs. 4, 2 vs. 4, and 2 vs. 3). Results showed that lizards were more accurate in discriminating sets of discrete items differing in number than the area of two individual items, in contrast to what had earlier been observed in spontaneous choice tests. Although we cannot exclude other factors that affected the performance of ruin lizards, the poor accuracy here observed in both experiments might reflect a true limit in lizards’ quantitative abilities.

## Introduction

Over the past decades, numerous studies have shown that many species are capable of discriminating whether one quantity is larger or smaller than another. This ability is highly adaptive in several contexts such as in choosing the larger group of potential mating partners, the larger amount of food, or the larger group of social companions to reduce predation risk (reviewed in Geary et al., 2014).

Two different methodological approaches have been used so far: spontaneous choice tests and training procedures (reviewed in Agrillo and Bisazza, 2014). The former method is used to investigate the spontaneous ability to discriminate between two quantities of biologically-relevant stimuli (e.g., food), whereas the latter method is used to assess the ability of animals to learn a numerical rule (e.g., select the larger set of neutral stimuli) in order to receive a reward. These methods differ in several respects. For instance, spontaneous choice tests simulate problems that animals can encounter in nature and the results obtained provide insight into the practical use of quantitative abilities in their habitat. On the contrary, in training procedures animals are tested in conditions that can be hardly compared to most of the problems faced in nature. However, spontaneous choice test presents a main limit: motivation. Indeed, lack of choice does not necessarily imply a lack of discrimination. For instance, if an animal does not show a preference between 3 and 4 fruits, this might occur because it is not motivated to select the larger set as both sets are large enough to satisfy it. To overcome this limitation, training procedures use neutral stimuli that allow to dissociate how large the sets are from how much food is consumed by the subject. As a consequence trained animals can also perform a larger number of trials compared to animals tested in spontaneous choice tests. For these reasons, the two methods are considered complementary and both are necessary to draw a firm picture of the quantificational abilities of a species (Agrillo and Bisazza, 2014).

Studies using both procedures have reported similarities in quantitative abilities in mammals, birds, amphibians, and fish. In these studies, accuracy decreases as the numerical ratio between two quantities increases (the value obtained by dividing the smaller quantity by the larger quantity) in agreement with Weber’s law (e.g., it is easier to discriminate 2 vs. 4 with a 0.5 ratio than 2 vs. 3 with a 0.67 ratio), thus suggesting the existence of a shared core number system among vertebrates inherited from a common ancestor (Feigenson et al., 2004; Beran, 2008). However, despite that quantitative abilities have been largely investigated in vertebrates, only a few data are available on reptiles (Burghardt, 1964; Soldati et al., 2017) and it is not clear whether their skills are comparable to those observed in the other taxa. Given the evolutionary significance of quantitative abilities there is no reason to believe that selective pressures in favor of processing quantitative information have not acted on this taxonomic group. As a consequence, studying reptilian quantitative abilities it is essential to gain a clear understanding of the evolution of cognition in vertebrates.

Recently, Miletto Petrazzini et al. (2017) found that ruin lizards (*Podarcis sicula*) were very accurate in discriminating between single food items (dead *Musca domestica* larvae) that differed in surface area up to 0.75 ratio (smaller surface area/larger surface area). However, lizards were not able to discriminate between two sets when food was presented in discrete quantities (e.g., 1 vs. 4 larvae; 0.25 ratio), contrary to data collected in mammals, birds, amphibians, and fish (Agrillo and Bisazza, 2014). These results represent an exception in the numerical cognition literature and suggest that quantitative abilities might reflect convergent evolution rather than a common origin.

The present study was designed to further investigate quantitative skills in ruin lizards using a training procedure. To this aim, we adapted a procedure recently developed with fish (Bisazza et al., 2014) in which guppies were trained on consecutive numerical discriminations of increasing difficulty by presenting fish with two groups of yellow disks differing in number. Here, we had two experiments. In Experiment 1, lizards were trained to discriminate between two sets containing a different number of items in order to obtain a food reward. In Experiment 2, lizards were presented with only two items differing in size to investigate their ability for discriminating between the areas of single entities. In both experiments, our goal was to assess the limit of the lizards’ discrimination capacity by presenting discriminations of increasing difficulty.

## Materials and Methods

### Subjects

A total of 21 adult ruin lizards, *Podarcis sicula* (14 subjects in Experiment 1 and 7 in Experiment 2) were used. Lizards were collected from the Province of Ferrara and were maintained at the Department of Life Sciences and Biotechnology, University of Ferrara. Lizards were group-housed in vivaria (115 cm × 35 cm × 48 cm) that were covered with sand on the bottom and contained hiding places (e.g., hollow bricks). The vivaria were maintained at 28 ± 2°C and were exposed to the natural photoperiodic conditions. The lizards were fed three times a week with dead *M. domestica* larvae and water was available *ad libitum*. During the experiments, the lizards were fed once a week at the end of the last day of testing. The lizards were kept in the laboratory for at least 1 month before being tested.

### Experimental Apparatus

**FIGURE 1:**
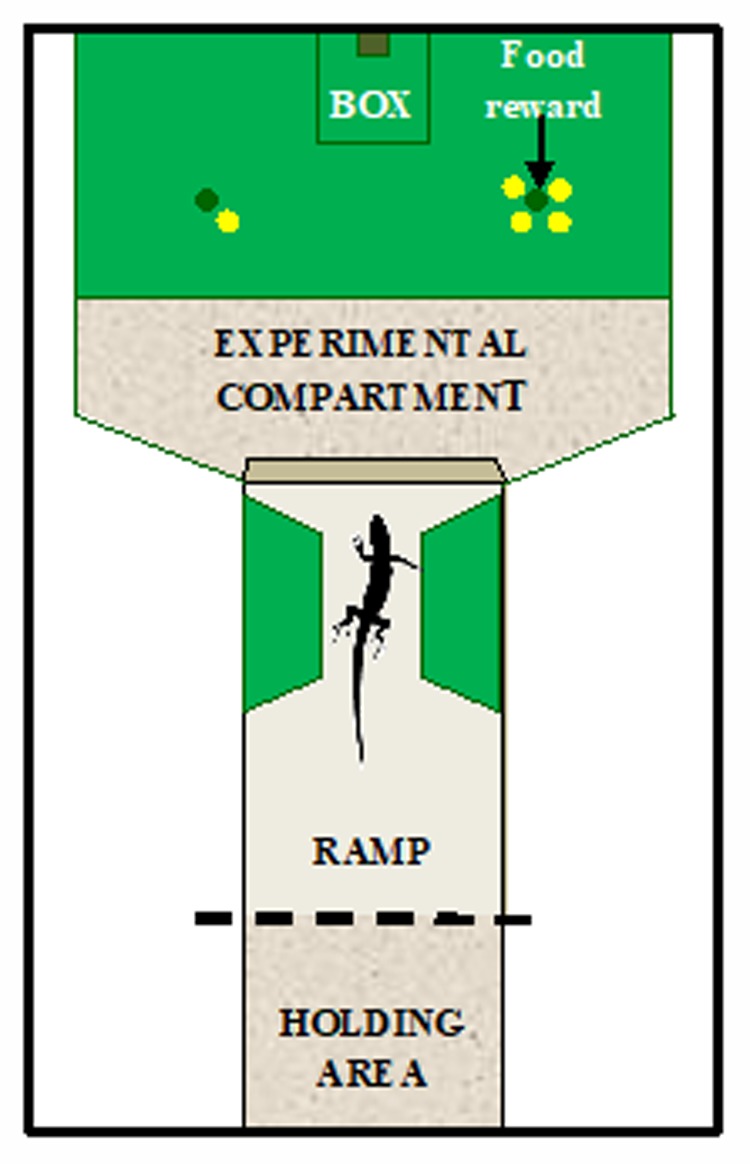
Schematic representation of the apparatus used in both experiments. The apparatus was divided in two compartments. One area, the tunnel, which served as holding area, and the other was the experimental compartment where a green plate was placed on the substratum. A ramp was inserted into the tunnel to allow access to the experimental compartment. Yellow disks were used as stimuli and were placed on the green plate. The food reward was hidden into the well in correspondence to the stimulus to be reinforced. Example of a trial of Experiment 1: lizards were trained to discriminate between 1 and 4 yellow disks in order to obtain a food reward.

The experimental apparatus was similar to that recently used to investigate spontaneous quantity discrimination in ruin lizards (Miletto Petrazzini et al., 2017). It consisted of a Y-shaped enclosure inserted into a sand-filled rectangular vivarium (60 cm × 40 cm × 22 cm) divided into an experimental compartment (30 cm × 30 cm) and a tunnel (30 cm × 18 cm) used as holding area where lizards were confined at the beginning of each trial to prevent them from viewing and entering the experimental compartment during the trial set-up (**Figure 1**). Lizards could view the stimuli from the top of a ramp inserted into the tunnel before entering the experimental compartment to make their choice. Two trapezoidal lateral compartments (12 cm × 7.5 cm × 6 cm) made of green plastic material narrowed the tunnel at the end of the ramp to keep the subjects in a central position and equidistant from the stimuli before choosing. Three 5-W fluorescent bulb lights illuminated the apparatus (1 light for the holding area and 2 lights for the experimental compartment). The experimental compartment contained an opaque green plastic plate (30 cm × 20 cm) containing 32 wells (diameter 1.5 cm, depth 0.5 cm). Green opaque plastic sheets (30 cm × 20 cm) with only two holes were used to cover all the wells except two during each trial. Twelve sheets with different spatial arrangements of the holes were used across trials to prevent the lizard from using spatial cues. Stimuli consisted of yellow plastic disks differing either in number (Experiment 1) or in size (Experiment 2) according to the schedule of each experiment.

A green plastic box (6 cm × 6 cm × 1 cm) with a handle (40 cm high) was placed adjacent to the bottom wall and in a central position.

To reduce the possibility of subjects using olfactory cues, the experimental compartment was saturated with the odor of eight non-visible larvae inserted in two boxes placed out of sight of the lizards.

All tests took place in a dark room maintained at a 28 ± 2°C. Trials were recorded by using a video camera (GZ-MG21E, JVC, Japan) suspended 1 m above the apparatus.

### Experimental Procedure

To reduce the potential effects of inter-individual variability, we used a within-subjects design to assess the effect of ratio in each experiment. However, to avoid any risk of interference between the tasks different lizards were tested in each experiment.

Two experiments were carried out with the same apparatus and basic procedure. Before the pre-training phase lizards underwent a 5-day acclimation phase. Each subject was individually inserted twice a day (once in the morning and once in the afternoon) into the holding area and was prevented from entering the experimental compartment by inserting a green plastic panel (18 cm × 35 cm) at the beginning of the ramp. After 5 s the panel was removed and the lizard was allowed to move around the entire apparatus for 15 min and eat one *M. domestica* larva placed next to one well in the experimental compartment. Six lizards (4 in Experiment 1 and 2 in Experiment 2) did not habituate to this procedure (i.e., did not move or moved but did not eat the larva) and were not admitted to the pre-training phase. As a consequence, the total sample consisted of 10 subjects in Experiment 1 and 5 subjects in Experiment 2.

During the pre-training phase, lizards underwent 12 trials in a single day (six trials in the morning and six in the afternoon). Each trial started with the subject in the holding area, the panel in place to block the view of the lizard and two groups of plastic disks, differing either in number or in size, placed next to two wells (**Figure 1**). During the first six trials, one larva was placed next to the larger stimulus. If the subject chose the wrong stimulus (by approaching it), it was allowed to then reach the correct one and eat the larva. During the last six trials, one larva covered with sand was inserted into the well in correspondence to the larger stimulus. Lizards could now only select one stimulus per trial. If the subject chose the correct stimulus it was allowed to eat the food reward while the other stimulus was gently covered with the green box. If the subject chose the wrong stimulus, the correct one was covered and the lizard did not receive any food reward. If the lizard did not make any choice within 10 min, the trial was considered invalid and repeated later. The left/right position of the larger stimulus was counterbalanced across trials. The distance from the ramp and the position of the stimuli on the panel were varied in each trial and were determined with a pseudo-random rule.

During the training phase, lizards received 12 trials per day divided into two sessions of six trials each (one session in the morning and one in the afternoon) for 5 days a week and for a maximum of 120 trials for each discrimination. The procedure was the same as that used during the last six trials of the pre-training phase.

In Experiment 1, lizards were trained to discriminate between two sets of equally sized yellow disks (diameter 1.5 cm, height 0.2 cm) differing in number. In this way, the cumulative surface area was congruent with the number of items (i.e., the stimulus with the larger number of disks was also larger in area). Each subject started with a 1 vs. 4 discrimination (0.25 numerical ratio) and proceeded to the next numerical discriminations, 2 vs. 4 (0.5 ratio) and 2 vs. 3 (0.67 ratio), only after they had reached a learning criterion of either 75% correct trials over two consecutive days or if the frequency of correct choices over the 120 trials was significant according to the chi-square test.

In Experiment 2, instead of presenting two differing quantities of disks, two single disks of differing sizes were presented. They varied in diameter from 1.5 to 3 cm, and each pair differed in surface area by a ratio of either 0.25, 0.50, or 0.67. These are the same ratios presented between the differing quantities of discrete items used in Experiment 1.

To further control for olfactory cues, all lizards except one underwent 70 more trials in which they were presented with two disks of equal size, but the food reward was hidden only in correspondence to one stimulus (randomly chosen).

## Results

In Experiment 1, 4 out of 10 lizards did not learn to discriminate 1 vs. 4 items and thus did not proceed to the next numerical discrimination. All of the remaining six lizards were able to discriminate 1 vs. 4 (**Table 1**). Three of the lizards reached the criterion of 75% correct responses in two consecutive days and their performance was above chance level. The performance of the other three lizards was above chance after 120 trials. These six subjects were then trained in the 2 vs. 4 discrimination but only one performed above chance level and reached the criterion of 75% correct responses in two consecutive days. This subject, however, failed to reach the criterion in the subsequent 2 vs. 3 discrimination (**Table 1**). We found no difference in the frequency of left–right correct choices in any lizard (chi-square test, all *p*-values > 0.05).

**Table 1:** Frequency of choices for the larger stimulus and chi-square test for Experiment 1 and Experiment 2.

Subject	Experiment	0.25	0.5	0.67
1	1	61/120 χ^2^= 0.03, *p* = 0.855		
2	1	61/120 χ^2^= 0.03, *p* = 0.855		
3	1	64/120 χ^2^= 0.53, *p* = 0.465		
4	1	68/120 χ^2^= 2.13, *p* = 0.144		
5	1	36/53 χ^2^= 6.81, *p* = 0.009	59/120 χ^2^= 0.03, *p* = 0.855	
6	1	45/69 χ^2^= 6.39, *p* = 0.011	62/120 χ^2^= 0.13, *p* = 0.715	
7	1	34/48 χ^2^= 8.33, *p* = 0.004	66/120 χ^2^= 1.20, *p* = 0.273	
8	1	74/120 χ^2^= 6.53, *p* = 0.011	62/120 χ^2^= 0.13, *p* = 0.715	
9	1	75/120 χ^2^= 7.50, *p* = 0.006	64/120 χ^2^= 0.53, *p* = 0.465	
10	1	77/120 χ^2^= 9.63, *p* = 0.002	21/31 χ^2^= 3.90, *p* = 0.048	57/120 χ^2^= 0.30, *p* = 0.584
11	2	54/120 χ^2^= 1.20, *p* = 0.273		
12	2	55/120 χ^2^= 0.83, *p* = 0.361		
13	2	59/120 χ^2^= 0.03, *p* = 0.855		
14	2	62/120 χ^2^= 0.13, *p* = 0.715		
15	2	63/120 χ^2^= 0.30, *p* = 0.584		

*All chi-squares have one degree of freedom.*

In Experiment 2, no lizard discriminated between two items differing by 0.25 ratio in area (**Table 1**), and, thus, did not proceed to the next discrimination. No left–right bias was observed (all *p*-values > 0.05).

In the control test for olfactory cues, the overall preference for the baited stimulus was not significant [mean ± SD: 0.54 ± 0.07; one sample *t*-test: *t*(13) = 2.047, *p* > 0.05].

Finally, in order to assess reliability of the scoring, 20% of the videos were independently coded by two observers. Reliability for choice was 100% (Pearson’s correlation *r* = 1, *p* < 0.001).

## Discussion

Quantitative abilities in animals have been extensively investigated using both spontaneous choice tests and training procedures (Agrillo and Bisazza, 2014).

In a recent study, Miletto Petrazzini et al. (2017) found that ruin lizards were spontaneously much more accurate in discriminating the size of two food items than their number. Here, we used a training procedure to further investigate quantitative abilities in ruin lizards. In particular, we adopted an extensive training procedure recently set up by Bisazza et al. (2014) using guppies that discriminated up to 4 vs. 5 disks, a much higher performance than the limit previously reported in this species.

In Experiment 1 of our study, six subjects out of 10 (60%) discriminated 1 vs. 4 items; among these, only one lizard learned a 2 vs. 4 discrimination. In contrast, in Experiment 2 no subject discriminated the area of two single items. These results differ from those obtained in the same species using a spontaneous food choice test (Miletto Petrazzini et al., 2017).

One possible explanation might be the existence of distinct quantification systems that are context-dependent, which serve to solve different problems and operate independently from each other (Feigenson et al., 2004). According to this view, there would be different quantitative systems for estimating food, conspecifics, and neutral stimuli. The different performance observed in our study and in Miletto Petrazzini et al.’s (2017) study might be due to the nature of the stimuli used (i.e., biological vs. non-biological) (Miletto Petrazzini et al., 2014). In line with this hypothesis, Piffer et al. (2013) showed that 1-week-old fish were able to discriminate between 7 and 14 objects, a capacity displayed in spontaneous choice tests in older fish (Bisazza et al., 2010) and Bánszegi et al. (2016) found that cats selected the larger set of food items when the ratio between the quantities was less than 0.5 whereas Pisa and Agrillo (2009) reported that cats can learn to discriminate 2 vs. 3 (0.67 ratio) geometric figures.

The overall performance observed in lizards was significantly lower compared to other vertebrates tested with training procedures (Agrillo and Bisazza, 2014) and, in particular, when compared to fish tested with the same methodological approach. Inter-specific differences could be due to a more general difference in learning abilities across species (Agrillo et al., 2012). However, this hypothesis seems to be unlikely as growing evidence suggests that reptiles possess learning abilities comparable to those observed in mammals (Wilkinson and Huber, 2012).

As an alternative, the poor performance here observed could reflect a true limit in lizards’ quantitative abilities. It has been argued that motivation plays a key – role in spontaneous choice tests and that the lack of choice in the presence of different quantities of items may simply reflect a lack of motivation rather than a lack of quantitative abilities. Conversely, the lack of discrimination after extensive training is likely to reflect a limit in the ability to process quantitative information instead of being the result of concomitant factors, such as motivation (Agrillo and Bisazza, 2014). Miletto Petrazzini et al. (2017) suggested that the lack of discrimination between multiple food items could be due to the foraging ecology of ruin lizards. Although in the present study we used neutral stimuli to overcome this potential limitation, lizards’ difficulty in discriminating quantities easily discriminated by other species with training procedures (Beran, 2006; Vonk and Beran, 2012; MacPherson and Roberts, 2013; Bisazza et al., 2014) could suggest that quantitative abilities in this species are limited. If so, it would raise an intriguing question as to whether quantitative skills of vertebrates have been inherited from a common ancestor or have independently developed as a result of convergent evolution.

Finally, we cannot exclude the possibility that the procedure used, although effective with guppies, is less suitable for lizards. It is worth noting that using the same methods for comparing different species may become a methodological weakness due to differences related to the species investigated (e.g., different ecological adaptations) (Agrillo et al., 2012). Guppies have a natural tendency to search for small yellow–orange fruits dropped into the river bottom and using a procedure close to the foraging habits of the species may have favored the achievement of finer numerical discriminations (Bisazza et al., 2014). Conversely, ruin lizards are mainly carnivorous and only occasionally eat small fruit. Hence we cannot exclude that, at least part of the poor performance observed in lizards, could be ascribed to motivational differences (i.e., the motivation of selecting groups of yellow objects may differ as a function of the diet).

One may argue that the results here obtained may be due to the small number of individuals tested, especially in Experiment 2. We are aware of the potential limits and the representativeness of the sample used to make inferences about populations. However, in training procedures, a small sample size is normally considered sufficient since animals undergo 100s of trials. For instance Pepperberg trained one parrot in several studies (reviewed in Pepperberg, 2012), Vonk and Beran trained three bears (Vonk and Beran, 2012), Perdue trained two African elephants (Perdue et al., 2012), and MacPherson and Roberts trained a single dog (MacPherson and Roberts, 2013). According to this view, the number of subjects tested in the present study would be enough to investigate quantitative abilities in lizards.

## Conclusion

The poor performance observed here using a methodological approach commonly used in other vertebrates, might suggest a limit in ruin lizards’ quantitative skills, although we cannot exclude other factors that affected their accuracy. Further investigation is now required using both the same and different procedures to replicate the results here obtained in order to reach a full understanding of quantitative abilities in reptiles, a vertebrate taxon underrepresented in the numerical cognition literature.

## Ethics Statement

This study was carried out in accordance with the recommendations of law of our country (Italy, D.L. 4 Marzo 2014, no. 26). The experimental protocol was authorized by the University of Ferrara Institutional Animal Care and Use Committee and by the Italian Ministry of Health (aut. no. 235/2015-PR).

## Author Contributions

MEMP, CB, and AF: developed the study concept, designed the experiments, and wrote the manuscript; MEMP: collected and analyzed the data. All authors agreed to be held accountable for this work.

## Conflict of Interest Statement

The authors declare that the research was conducted in the absence of any commercial or financial relationships that could be construed as a potential conflict of interest.
